# Interventions and impact of pharmacist-delivered services in perioperative setting on clinically important outcomes: a systematic review and meta-analysis

**DOI:** 10.1177/20420986241260169

**Published:** 2024-07-31

**Authors:** Lina Naseralallah, Somaya Koraysh, Bodoor Aboujabal, May Alasmar

**Affiliations:** Department of Pharmacy, Hamad Medical Corporation, Doha, Qatar; School of Pharmacy, Institute of Clinical Sciences, Sir Robert Aitken Institute for Medical Research, University of Birmingham, Birmingham, UK; Department of Pharmacy, Hamad Medical Corporation, Doha, Qatar; Department of Pharmacy, Hamad Medical Corporation, Doha, Qatar; College of Medical, Veterinary and Life Sciences, University of Glasgow, Glasgow, UK; Department of Pharmacy, Hamad Medical Corporation, Doha, Qatar

**Keywords:** clinically important outcomes, length of stay, meta-analysis, perioperative settings, pharmaceutical care, pharmacist, quality of care, readmission, systematic review

## Abstract

**Background:**

The perioperative arena is a unique and challenging environment that requires coordination of the complex processes and involvement of the entire care team. Pharmacists’ scope of practice has been evolving to be patient-centered and to expand to variety of settings including perioperative settings.

**Objectives:**

To critically appraise, synthesize, and present the available evidence of the characteristics and impact of pharmacist-led interventions on clinically important outcomes in the perioperative settings.

**Design:**

A systematic review and meta-analysis.

**Methods:**

We searched PubMed, Embase, and CINAHL from index inception to September 2023. Included studies compared the effectiveness of pharmacist-led interventions on clinically important outcomes (e.g. length of stay, readmission) compared to usual care in perioperative settings. Two independent reviewers extracted the data using the DEPICT-2 (Descriptive Elements of Pharmacist Intervention Characterization Tool) and undertook quality assessment using the Crowe Critical Appraisal (CCAT). A random-effect model was used to estimate the overall effect [odds ratio (OR) for dichotomous and standard mean difference (SMD) for continuous data] with 95% confidence intervals (CIs).

**Results:**

Twenty-five studies were eligible, 20 (80%) had uncontrolled study design. Most interventions were multicomponent and continuous over the perioperative period. The intervention components included clinical pharmacy services (e.g. medication management/optimization, medication reconciliation, discharge counseling) and education of healthcare professionals. While some studies provided a minor description in regards to the intervention development and processes, only one study reported a theoretical underpinning to intervention development. Pooled analyses showed a significant impact of pharmacist care compared to usual care on length of stay (11 studies; SMD −0.09; 95% CI −0.49 to −0.15) and all-cause readmissions (8 studies; OR 0.60; 95% CI 0.39–0.91). The majority of included studies (*n* = 21; 84%) were of moderate quality.

**Conclusion:**

Pharmacist-led interventions are effective at improving clinically important outcomes in the perioperative setting; however, most studies were of moderate quality. Studies lacked the utilization of theory to develop interventions; therefore, it is not clear whether theory-derived interventions are more effective than those without a theoretical element. Future research should prioritize the development and evaluation of multifaceted theory-informed pharmacist interventions that target the whole surgical care pathway.

## Plain language summary

The impact of pharmacist activities on clinical outcomes in perioperative settings

Why was the study done? The time around the surgery imposes significant risks to patient’s health. While technical aspects of a procedure are important, it should be combined with the provision of optimal healthcare quality to increase the likelihood of desired clinical outcomes. Pharmacists are effective healthcare team members who have the potential to improve patient’s outcomes in the perioperative settings. It is hence imperative to explore the roles and impact of clinical pharmacists in these settings. Thus far, there is no synthesis of literature regarding the pharmacist roles and effectiveness in the perioperative setting. What did the researchers do? We aimed to summarize and appraise the quality of evidence on the characteristics and impact of pharmacist activities on clinical outcomes in the perioperative settings. Three library databases were examined to identify studies eligible for inclusion. Two authors extracted data and assessed the quality of included studies. Statistical analysis was used to look at the success of the pharmacist interventions on different endpoints. What did the researchers find? A total of 25 studies were included. Most pharmacist activities consisted of multiple components (such as medication management and educating other healthcare providers) and spanned the whole perioperative journey. The analysis of included studies showed that pharmacist activities reduced the elapsed time in hospital and the number of times a patient winds up going back to a hospital after discharge. Most studies did not discuss the methods adopted to develop pharmacist activities. What do the findings mean? A number of pharmacist activity types were shown to be successful in reducing the duration of hospitalization and readmission episodes. New directions for future research should investigate the development of pharmacist-led interventions in terms of structure and processes to ensure the reproducibility of these interventions.

## Introduction

The perioperative period is defined as the time lapse surrounding the surgical act which can be subdivided into three stages: preoperative, intraoperative, and postoperative.^1,2^ The perioperative environment is known to be one of the most complex and challenging areas within a hospital with significant safety risks.^3^ Studies showed that half of all adverse events in hospitals occur in perioperative settings.^4,5^ Therefore, the World Health Organization launched the campaign ‘Safe Surgery Saves Lives’ in 2008 which aims to improve the safety of surgical procedures and implement patient safety best practices to reduce the incidence of adverse events both in the operating room and in the ward.^6^ For decades, the objective of safe surgery was centered on technical procedures. However, there is emerging evidence that improving access to surgical healthcare alone does not result in improved health outcomes unless it is coupled with quality perioperative care.^7^ Thus, in recent years, much attention has been devoted to the empowerment of nontechnical skills and interpersonal communication which contribute to the improvement of patient-centered quality metrics and subsequently to reducing harm in perioperative settings.^1,6^

Multidisciplinary teams have been proposed as an effective approach to enhance the quality and safety of care in perioperative settings and to reinforce the importance of patient-centeredness.^3^ While the roles and outcomes of most healthcare providers in these settings are well established in the literature, the roles and impact of pharmacist integration into perioperative settings is seldom examined.^8–10^ Over the past few decades, the scope of practice for pharmacists has been evolving, which enabled them to embrace new roles in a variety of settings. Pharmacists’ expanded professional role includes variety of activities such as medication reviews, medication therapy management, patient counseling, independent prescribing, telepharmacy services, vaccine dissemination, community education, and emergency preparedness and response.^11–13^ The American Society of Health-System Pharmacists highlighted the greater demand for increased pharmacy involvement in the perioperative medication-use process by incorporating additional activities.^14^ Clinical pharmacists could have unique roles in the perioperative settings owing to their advanced therapeutic knowledge and experience which make them well poised to add value to emergency response efforts as paralleled in other settings.^14,15^ This could potentially lead to a significant improvement to patient care in terms of both efficacy and safety.^15^

Outcome indicators are increasingly used in quality measurement programs in various countries to monitor and compare hospital performance, with the aim of identifying areas for improvement. Three outcome measures that are frequently used to evaluate quality of care in hospitals are in-hospital mortality, readmission rate, and length of stay (LOS).^16–18^ LOS is considered a pivotal clinical outcome metric used as a proxy of efficient hospital and healthcare systems management. Prolonged LOS not only decreases reimbursement margins but is also associated with negative patient and staff experience, as well as increased inpatient complications (e.g. falls, adverse drug events, hospital-acquired infections).^19–22^ Similarly, decreasing the rate of hospital readmissions has been targeted as a high priority for hospitals across the globe, hastened by implementation of public reporting and financial penalties for excess readmissions.^23,24^ Furthermore, to obtain a comprehensive picture of quality, it is attractive to jointly report outcome measures to ensure consistent improvement in all outcomes of interest. For instance, mortality may be very low in some patient groups but readmission rate is high.^25^

It is hence imperative to explore the roles and impact of pharmacists on these clinical outcomes in perioperative settings. This systematic review and meta-analysis aimed to critically appraise, synthesize, and present the available evidence of the characteristics and impact of pharmacist-led interventions on clinically important outcomes in the perioperative settings.

## Methods

This systematic review and meta-analysis was conducted utilizing the Preferred Reporting Items for Systematic Reviews and Meta-Analyses (PRISMA) guideline (Supplemental Materials 2, see ESM)^26^ and is registered prospectively in the International Prospective Register of Systematic reviews (PROSPERO): (CRD42023460812).

### Literature search

We searched the following databases from index inception until September 2023: PubMed, Embase, CINAHL, and Google Scholar. The literature search was carried out using natural language keywords and, where applicable, MeSH terms and EMTREE (controlled vocabulary thesaurus) for data search in PubMed and Embase, respectively. Each database was searched using variants of keywords such as pharmacist, pharmacy, pharmaceutical care, perioperative period, perioperative care, surgery, and procedure (Supplemental Materials 1, see ESM). The reference lists of included studies were manually reviewed to search for any additional studies.

### Types of studies and eligibility criteria

Studies were included if they were (1) randomized controlled trials (RCTs), quasi-experimental, pre-post-, prospective, and retrospective cohort; (2) evaluated a clinical pharmacist-led intervention; (3) conducted in the perioperative period; (4) had a control or comparison group (with healthcare professionals other than a pharmacist); (5) measured any of the following clinically important endpoints [LOS, readmission, mortality, emergency department (ED) visits, unplanned outpatient visits, any healthcare encounter]; (6) published in a peer-reviewed journal in English or Arabic languages and available in full text. Case reports, expert opinions, systematic reviews, letters to editors, commentaries, correspondences, news articles, and qualitative studies were excluded from this review, as were conference abstracts if not available in full text. We also excluded studies focusing on pediatric patients.

### Study selection

The articles found in the database search were transferred to Rayyan, a web application for systematic reviews, to identify and delete any duplicated articles.^27^ Two authors (LN, SK) independently screened the titles and abstracts of all retrieved studies. Then, full-text screening of each potentially eligible study was independently done by two reviewers (LN and SK or BA and MA), and discrepancies were resolved through consensus. If the difference remained unresolved, a third reviewer adjudicated to reach a predefined consensus.

### Data extraction

A bespoke data extraction tool was developed based on the Descriptive Elements of Pharmacist Intervention Characterization Tool (DEPICT-2).^28^ DEPICT-2 is a validated instrument for accurately describing and characterizing the details of pharmacist interventions. The tool consists of 93 items, subsumed into 11 domains: contact with recipient, setting, target population, clinical data sources, variables assessed, pharmacist intervention, timing of intervention, material that support intervention, repetition, communication with recipient, and changes in therapy and laboratory tests.^28^ The final data extraction sheet included the following components:

- *General information*: author(s), year, country, study design, objectives, population, sample size, study duration, and surgical unit(s).
- *Description of intervention*: recipients, focus of intervention, setting, method of communication, clinical data source, pharmacist action, timing and frequency of action, and materials that support action.
- Key findings.

The final data extraction sheet was piloted on four studies prior to its use. An independent, duplicate data extraction of each study was undertaken (LN, SK, MA, or BA).

### Quality assessment

Quality assessment of the selected articles was undertaken by independent reviewers working in pairs (LN, SK, MA, or BA) using the validated Crowe Critical Appraisal tool (CCAT) version 1.4.^29^ The CCAT contains 22 items grouped into 8 categories and is applicable to all study designs, with the highest possible score being 40. The tool helps in recording scores for each category so that the final score is not influenced by an overall opinion about the study. The quality of studies was categorized as follows: high quality (36 and above), moderate quality (30–35), and low quality (29 and below). This was based on a consensus reached by the reviewers to group studies by quartiles, a similar approach adopted by Donnelly *et al.*^30^ and El-Awaisi *et al*.^31^ The author of the CCAT tool was also contacted to ensure that this method of interpretation was valid.

### Outcome measures

Impact of clinical pharmacist interventions on clinically important outcomes in the perioperative setting was the primary measure of interest identified in included studies. Clinically important outcomes include any of the following endpoints: LOS, readmission, mortality, ED visits, unplanned outpatient visits, and any healthcare encounter.

The secondary outcome was to describe the adopted clinical pharmacist interventions in accordance with the DEPICT-2 tool to assess the following domains: target population, setting where the intervention took place, contact with recipients, pharmacist activities, source of guide for the intervention, and materials that support intervention.

### Data analysis

Our analysis included studies that reported at least one of the following clinically important outcomes: healthcare utilization (LOS, readmission, mortality, ED visit, and any healthcare encounter) and mortality compared with usual care. Where adequate data for the meta-analysis were reported, the odds ratios (ORs) for dichotomous outcomes or standard mean differences (SMDs) for continuous outcomes, both with 95% confidence intervals (CIs) and two-sided *p* values for each outcome, were derived. Statistical heterogeneity between studies was quantified using the Chi-squared test and the *I*^2^ statistic. A random-effects model was used to calculate the pooled effect estimate for outcomes as heterogeneity (*I*^2^ > 50%) was expected due to the variation in the interventions and patient populations. We evaluated publication bias by inspection of funnel plot and Egger’s regression test. In all analyses, significance was established at values of *p* < 0.05. Data analyses were performed using IBM Statistical Package for Social Sciences (IBM SPSS Statistics, Version 29.0; IBM Corp, Armonk, NY, USA).

## Results

### Search results and study selection

**Figure 1.**
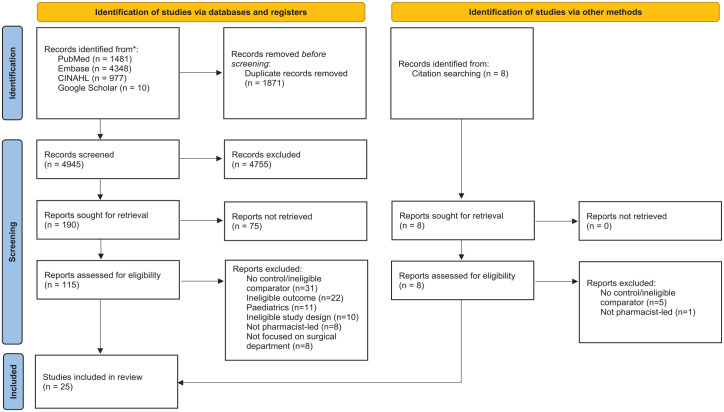
PRISMA chart describing study retrieval and selection. PRISMA, Preferred Reporting Items for Systematic Reviews and Meta-Analyses.

A total of 6816 potential articles were identified from searches in the electronic databases, and 8 additional records were identified in reference lists of included studies (Figure 1). Of these, 115 were reviewed in full text, and 25 were included.

### Description of included studies

#### Countries and surgical departments

The majority of studies were conducted in the United States (*n* = 9; 36%)^32–40^ and China (*n* = 9; 36%),^41–49^ followed by the United Kingdom (*n* = 3; 12%),^50–52^ and one (4%) each in Australia,^53^ Netherland,^54^ Pakistan,^55^ and Sudan^56^ (Table 1). Most of the studies (*n* = 8; 32%) were carried out in orthopedic surgery,^35,37,38,41,44,47,49,52^ followed by five (20%) studies each that included multiple units^43,53–56^ and general surgery^39,42,46,50,51^ (Table 1). Three studies (12%) were conducted in bariatric surgical units,^33,34,40^ and two (8%) each in cardiothoracic^36,48^ and transplant units.^32,45^ The majority of studies were published in 2019 onward, except for one each in 2015,^54^ 2016,^48^ and 2018.^38^

**Table 1.** Characteristics of included studies.

Study	Country	Objective	Study design	Sample size	Study duration	Surgical unit	Included patient characteristic	Follow-up duration	Findings of interest
Alsheikh *et al.* (2020)	USA	To evaluate the impact of clinical transplant pharmacy services on the kidney transplant program in terms of inpatient LOS and all-cause 30-day readmission rates	Retrospective cohort study	205 patients (101 preintervention, 104 postintervention)	2 years	Transplant	Patients who received a kidney transplant	30 days	- LOS (day): 6.58 (SD 5.27) *versus* 5.76 (SD 4.31); *p* = 0.041, 95% CI (−0.501 to 2.150) - 30-day readmission: 36 (35.6%) *versus* 27 (25.9%); *p* = 0.133
Bansal *et al*. (2019)	UK	To assess the implementation of the ESMOS and to evaluate its impact on postoperative care outcomes at 12 months after it was rolled out	Retrospective cohort study	246 patients	1 year	General (hepato-pancreato-biliary, upper and lower GI, vascular)	Patients who underwent major elective general surgical procedures with an ASA score for physiological status of more than or equal to 2 (i.e. a patient with a mild systemic disease)	12 months	- LOS (day) in the lower GI (median reduction: −2; IQR: −4, 1.8; *p* = 0.038), HPB (median reduction: −4.5; IQR: −7, −1; *p* = 0.001), and vascular (median reduction: −2; IQR: −4, 0; *p* = 0.043) - Median LOS was longer than median expected LOS in the upper GI specialty (median reduction: 5; IQR: −3, 17; *p* = 0.055) -Total LOS reduction in patients whose LOS fell in the 25th to 75th percentile range was −96 days in lower GI, −81 days in HPB, and −104 days in vascular subspecialties, which summed up to −281 days
Bansal *et al*. (2019)	UK	To evaluate the impact pharmacist involvement can have on enhanced recovery pathways with a focus on medicines optimization specifically to reduce the LOS and incidence of postoperative complications	Prospective before and after study	100 patients (50 pre intervention, 50 post intervention)	3 years	Colorectal	Patients undergoing elective colorectal surgery with an expected LOS of at least one night and with an ASA score of more than or equal to 2	4 months	- LOS (day): 10.5 *versus* 7.5; *p* < 0.001 - 30-day readmission: 9 *versus* 7; *p* > 0.05
Butt *et al*. (2019)	Pakistan	To evaluate the impact and cost-benefit value of pharmacist’s educational intervention for antibiotic use in postsurgical prophylaxis	Prospective quasi-experimental study	450 patients (225 control, 225 intervention)	Not reported	Multiple (general, orthopedic, gynecology)	Patients with clean/clean-contaminated surgeries from three different surgery wards, general, orthopedic, and gynecology on surgical prophylaxis and without systemic disease	Not reported	- LOS (day): 5.4 (SD 4.814) *versus* 4.50 (SD 3.398); *p* = 0.023
Elnour *et al.* (2022)	Sudan	To test that the clinical pharmacist’s interventions may facilitate the implementation of SAP protocol and subsequent reduction of surgical site infections	RCT	226 patients (113 control, 113 intervention)	Not reported	Multiple (hernia repair, thyroidectomy, appendectomy, cholecystectomy)	Patients of both genders above 18 years and less than 65 years undergoing elective surgery	14 days	- LOS for >4 days: 3.5% *versus* 1.7%; *p* < 0.001 - LOS 1, 2, 2–4 days were not significant
Falconer *et al*. (2021)	USA	To evaluate the feasibility and effectiveness of a pharmacy-led initiative for facilitating discharge medicine reconciliation after bariatric surgery	Retrospective cohort study	353 patients (158 pre intervention, 195 post intervention)	Not reported	Bariatric	Patients aged 18 years or older who underwent primary or revisional laparoscopic or robotic weight loss surgery	30 days	- 30-day readmission: 12 (7.6%) *versus* 3 (1.5%); *p* = 0.04 - Outpatient medication-related phone calls: 49 (31%) *versus* 76 (39%); *p* = 0.04
Fitzpatrick *et al*. (2023)	UK	To investigate the significance of pharmacist input, the impact on reducing prescribing errors, postoperative patient outcomes, and patient and staff satisfaction with the service	Retrospective pre/post intervention implementation	209 patients (80 preintervention, 129 postintervention)	20 weeks	Orthopedic	Patients undergoing arthroplasty TKR/UKR or THR	9 days	- LOS (day): 0.99 (SD 0.57) *versus* 1.1 (SD 0.48); *p* = 0.16 -Any healthcare encounter (9 days follow-up): 52.5% *versus* 36.9%; *p* = 0.03 - 100% of patients felt that the pharmacist helped them understand the need for blood thinners and understand what tablets should be withheld before surgery - 73% of patients with history of orthopedic surgery found the experience ‘much better’ - 87% felt the consultation provided the correct amount of information - 4.79% of staff felt the service freed up time for them to spend on clinical care of patients - 100% of staff felt the service improved efficiency of medicine supply and improved prescribing standards
Han *et al*. (2022)	USA	To evaluate the impact of a bariatric clinic-based pharmacist on inpatient LOS, medication errors, and patient experience	Retrospective cohort study	135 patients (67 control, 68 intervention)	Not reported	Bariatric	Patients admitted for primary bariatric surgery	Not reported	- LOS (h): 57.9 *versus* 55.5; *p* = 0.56. There was no statistically significant difference by procedure type. - Over 90% of respondents strongly agreed or agreed to the benefits of an in-clinic pharmacist consultation in the areas of pharmacist clarity, pharmacist answer quality, pharmacist helpfulness, and self-preparedness - Overall satisfaction with the pharmacist consultation, 97% of patients reported ‘strongly agree’ or ‘agree’
Hyland *et al*. (2020)	USA	To assess the impact of an orthopedic clinical pharmacist service on institutional TJA complication rates and costs	Prospective, interventional, sequential cohort study	1227 patients (694 baseline, 533 post implementation)	20 months	Orthopedic	Patients undergoing TKR or THR	30 days	- LOS (day): 2.9 *versus* 2.8 - 30-day readmission: 4.8% *versus* 1.3%; *p* = 0.002, RR 0.28, 95% CI (0.12–0.67) -Patients who interacted with a pharmacist exhibited a higher degree of self-rated understanding of medications (93.74% *versus* 75.00% with scores ⩾4 out of 5) - 12.90% of the patients who did not interact with the pharmacist had questions about their medications, as compared to only 2.04% of the patients who interacted with a pharmacist - Of the 49 patients who interacted with a pharmacist, 100% of these patients reported they found the service valuable
Kwiatkowski *et al*. (2021)	USA	To implement and evaluate a pharmacist-led BLA clarification interview service in the preoperative setting	Quasi-experimental	87 patients (50 control, 37 intervention)	5 months	Cardiothoracic	Patients with BLA, perioperative clinic appointment, and surgery requiring beta lactam as prophylaxis	30 days	- LOS (day): of 2 (IQR 2.0–4.5) *versus* 2 (IQR 2.0–2.0); *p* = 0.014
Nguyen *et al*. (2020)	Australia	To evaluate the impact of a PREP pharmacist on postoperative medication management	Randomized prospective interventional study	104 patients (51 control, 53 intervention)	3 months	Multiple (general, vascular, orthopedic, gynecological, and urology)	Patients for elective surgery at high risk for medication misadventure	Duration of hospitalization	- Patient discharged from hospital by midday: 14% *versus* 25%; *p* = 0.247
Patel *et al*. (2022)	USA	To evaluate the effect of the institution’s new standardized postoperative multimodal pain regimen guidance on cumulative opioid prescribed postoperatively at discharge, as measured in MED	Retrospective cohort study	92 patients (40 pre intervention, 52 post intervention)	8 months	Orthopedic	Patients 18 years or older who underwent TKR or THR	6 months	- LOS (day): 2.0 (IQR 1.3–3.8) *versus* 1.5 (IQR 1.0–3.0); *p* = 0.04
Shang *et al*. (2021)	China	To assess the impact of clinical pharmacist services on the use of anticoagulant drugs, the rationality of medication and the incidence of thrombosis in patients with TJA	Retrospective cohort study	577 patients (240 baseline, 377 intervention)	36 months	Orthopedic	Patients undergoing THR or TKR	36 months	- LOS (day): 20.03 (SD 9.21) *versus* 15.20 (SD 5.57); *p* = 0.000
Smith *et al*. (2018)	USA	To determine whether a pharmacist-led, patient-directed intervention can reduce opioid use following THR or TKR	Randomized, pragmatic clinical trial	561 patients (286 usual care, 275 intervention)	Not reported	Orthopedic	Patients 20 years and older who were scheduled to undergo TKR or THR in the subsequent 14 to 21 days. Patients were ranked according to predicted risk of persistent opioid use and the top 60% were selected	90 days	- Count of face-to-face office visits, mean (95% CI): 8.17 (7.48–8.86) *versus* 8.35 (7.62–9.08), crude difference: 0.18 (−0.82 to 1.18), adjusted rate ration: 1.04 (0.94–1.16) - Count of telephone encounters, mean (95% CI): 7.33 (6.59–8.07) *versus* 7.93 (7.01–8.85), crude difference: 0.60 (−0.58 to 1.77), adjusted rate ratio: 1.11 (0.98–1.27) - Count of email encounters, mean (95% CI): 2.03 (1.69–2.38) *versus* 2.07 (1.68–2.47), crude difference: 0.04 (−0.48 to 0.56), adjusted rate ratio: 0.95 (0.72–1.25) - Count of office visits with KPNW ED or urgent care, mean (95% CI): 0.21 (0.14–0.28) *versus* 0.16 (0.11–0.22), crude difference: −0.05 (−0.14 to 0.04), adjusted rate ratio: 0.94 (0.53–1.66) - Count of office visits with non-KPNW ED, mean (95% CI): 0.05 (0.01–0.08) *versus* 0.02 (0.00–0.04), crude difference: −0.03 (−0.07 to 0.01), adjusted rate ratio: 0.46 (0.04–4.07)
Smith *et al*. (2023)	USA	To evaluate the differences between mean blood glucose levels, glucose values within goal range, and postoperative outcomes between a clinical pharmacist-driven glycemic control *versus* standard care	Retrospective cohort study	186 patients (66 standard care, 120 intervention)	16 months	Colorectal	Patients with type 2 diabetes and HbA1c <8%	Duration of hospitalization	-LOS (day): 5.9 (SD 4.9) *versus* 4.4 (SD 2.9); *p* = 0.046
SUREPILL Study Group, 2015	Netherlands	To evaluate ward-based pharmacy interventions to reduce medication-related harm in surgical patients	RCT	1094 patients (547 control, 547 intervention)	2 years	Multiple (GI, hepato-pancreato-biliary, vascular)	Patients admitted for elective surgery with expected hospital stay longer than 48 h	Duration of hospitalization	- LOS (day): 9 (IQR 6–13) *versus* 8 (IQR 6–12); *p* = 0.066 - 3-months readmissions: 64/362 (17.7%) *versus* 84/362 (23.2%); *p* = 0.063 - QoL: QoL EQ-5D: 0.81 (0.73–1.00) *versus* 0.81 (0–69–1.00); *p* = 0.337. QoL EQ-VAS: 70 (60–80) *versus* 70 (60–80); *p* = 0.102
Tong *et al*. (2022)	China	To evaluate the role of pharmacists in the postoperative complications and nutritional status of perioperative patients with colorectal cancer	Retrospective pre/post cohort study	284 patients (137 preintervention, 147 postintervention)	Not reported	Colorectal	Patients with pathologically diagnosed colon/rectal cancer	Duration of hospitalization	- LOS (day): 10.3 *versus* 11.0; *p* = 0.110
Van Prooyen *et al*. (2023)	USA	To evaluate the impact of an inpatient pharmacy consult on discharge medication doses, classes, and formulations prescribed for patients after bariatric surgery	Retrospective-prospective intervention study	252 patients (167 control, 85 intervention)	6 months	Bariatric	Patients 18 years or older and admitted to the hospital for weight loss bariatric surgery with either RYGB, SG	30 days	- 30-day readmission: 4.2% *versus* 3.5%; *p* = 1 - ED visits within 30 days: 2.4% *versus* 5.9%; *p* = 0.28
Wang *et al*. (2020)	China	To evaluate the effects of the CPGT on the improvement of PONV and prophylaxis administration	Prospective before and after study	156 patients (82 preintervention, 74 postintervention)	1 year	Multiple (abdominal surgery)	Female patients with ASA grades of 1–3 scheduled for abdominal surgery under general anesthesia	Duration of hospitalization	- LOS (day): 5.27 (SD 5.04) *versus* 3.88 (SD 3.62); 95% CI: −0.504 to 0.310; *p* = 0.639
Wang *et al*. (2023)	China	To construct a perioperative pharmaceutical care model and clinical pathway for patients undergoing orthopedic surgeries and assess their impact on pain management	Before and after study	320 patients (158 control, 162 intervention)	1 year	Orthopedic	Patients 18 years or older with elective or limited term orthopedic surgery	Not reported	- LOS (day): 12.29 (SD 5.96) *versus* 9.97 (SD 4.6); *p* = 0.001 - LOS in patients with expected severe postoperative pain: 14.4 ± 5.7 *versus* 13.2 ± 4.1; *p* = 0.507
Yang *et al*. (2019)	China	To comprehensively assess the impact of pharmacist-led posttransplant medication management for kidney transplant recipients	Retrospective cohort study	204 patients (84 pre intervention, 120 post intervention)	2 years	Transplant	Patients receiving living-donor or deceased-donor kidney transplants	30 days	- Readmission rates within 7 and 30 days were 0% in both groups
Zhang *et al*. (2021)	China	To evaluate the clinical effects of a clinical pharmacist intervention on inappropriate proton pomp inhibitor prescriptions in a tertiary general hospital hepatobiliary surgery ward	Retrospective pre/post intervention study	717 patients (420 pre intervention, 297 post intervention)	6 months	Hepatobiliary	Patients receiving proton pomp inhibitor	Duration of hospitalization	- LOS (day): 10.83 (SD 8.75) *versus* 9.95 (SD 7.62); *p* = 0.162
Zheng *et al*. (2022)	China	To investigate the impact of medication reconciliation, through avoidance of unintentional medication discrepancies, on enhanced recovery after surgery programs designed for older patients undergoing orthopedic joint surgery	RCT	65 patients (32 control, 33 intervention)	Not reported	Orthopedic	Patients who underwent elective orthopedic joint surgery who had PJI and were scheduled for two-stage revision	30 days	- LOS (day) for the first stage: 20.7 *versus* 16.3; *p* = 0.03 - 30-day readmission: 3 *versus* 0; *p* > 0.05 - Unplanned outpatient visits within 30 days of discharge: 4 *versus* 0; *p* > 0.05 - Perioperative pain management: 6.2 ± 1.8 points *versus* 8.4 ± 1.8 points - Management of nausea and vomiting: 6.7 ± 4.6 points *versus* 8.9 ± 2.1 points - Enough information received and feeling of readiness at discharge: 7.7 ± 1.6 points *versus* 9.7 ± 1.8 points
Zhou *et al*. (2016)	China	To study the impact of multifaceted pharmacist interventions on antibiotic prophylaxis in patients undergoing clean or clean-contaminated operations in cardiothoracic department	Pre-post quasi-experimental study	963 patients (412 baseline, 551 intervention)	2 years	Cardiothoracic	Patients undergoing cardiothoracic surgery and the wound class of the surgical operation was clean or clean-contaminated	Not reported	- LOS (day): 23.3 (SD 8.9) *versus* 20.9 (SD 8.9); *p* = 0.001
Zhou *et al*. (2023)	China	To evaluate the clinical effects and cost-effectiveness of pharmacist-led intervention in the perioperative anti-infection prophylaxis of patients undergoing orthopedic internal fixation	Retrospective cohort study	472 (236 control, 236 intervention)	1 year	Orthopedic	Patients with elective internal fixation surgery and with a wound class categorized as clean	6 months	- LOS (day): 11.17 (SD 3.15) *versus* 10.24 (SD 2.93); *p* = 0.012, reduced by 0.71 - 6-Month readmission 3.8% *versus* 5.5%; *p* = 0.338

ASA, American Society of Anesthesiologists; BLA, beta-lactam allergy; CI, confidence interval; CPGT, clinical pharmacist-led guidance team; ED, emergency department; EQ-5D EuroQOL-5 dimension questionnair; ESMOS, Enhanced Surgical Medicines Optimization Service; GI, gastrointestinal; HPB, hepato-pancreato-biliary; IQR, interquartile range; KPNW, Kaiser Permanente Northwest; LOS, length of stay; MED, morphine equivalent dose; PJI, periprosthetic joint infection; PONV, postoperative nausea and vomiting; PREP, PeRiopErative and Prescribing; QoL, quality of life; RCT, randomized controlled trial; RR, relative risk; RYGB, Roux-en-Y gastric bypass; SAP, surgical antimicrobial prophylaxis; SD, standard deviation; SG, sleeve gastrectomy; SMD, stndard mean difference; SUREPILL, Surgery and Pharmacy in Liaison; THR, total hip replacement; TJA, total joint arthroplasty; TKR, total knee replacement; UKR, unicompartmental knee replacement.

#### Study design

The majority of studies (*n* = 20; 80%) used an uncontrolled study design, 12 (48%) retrospective,^32–34,37,39,41,42,45,46,49,51,52^ 5 (20%) prospective,^35,36,43,50,55^ 3 (12%) retro-prospective studies,^40,44,48^ with a variety of observational methodological designs (Table 1). The remaining five (20%) were controlled, all of which were randomized.^38,47,53,54,56^ Follow-up duration among the included studies ranged from 9 days^52^ to 36 months.^41^

#### Study population

The studies’ sample sizes ranged between 65 ^47^ and 1227 patients^35^ (Table 1). Other than specifying one or more surgical units, the majority of studies (*n* = 13; 52%) focused on a particular class of medication including antibiotics (*n* = 5; 20%),^36,48,49,55,56^ analgesics (*n* = 3, two of which on opioids),^37,38,44^ and one each on anticoagulants,^41^ antidiabetic drugs,^39^ antiemetics,^43^ proton pump inhibitor,^46^ and total parenteral nutrition (TPN)^42^ (Table 2). Among the reminder studies, two focused on patients with mild systemic disease^50,51^ and two on patients at higher risk for medication misadventures.^38,53^ One study included female patients only as they are at higher risk for postoperative nausea and vomiting^43^ (Table 1).

**Table 2.** Description of pharmacist intervention according to DEPICT version 2.

Study	Recipients	Focus of intervention	Mode of contact with the recipient	Setting where recipient received intervention	Methods of communication	Clinical data sources	Source of guide for intervention	Pharmacist action(s)	Timings of pharmacists’ action	Frequency of contacts	Materials that support action(s)
Alsheikh *et al.* (2020)	Patients and surgeons	All medications with focus on immunosuppressants	Contact with group (rounds), one-on-one (clinic)	Hospital (bedside and outpatient clinic)	Face to face, telephone	Medication chart/patient history taking	CMS clinical guidelines	1. Prekidney transplant evaluation 2. Daily rounds on admitted patients to monitor therapy 3. Daily meetings with the transplant multidisciplinary team to discuss the therapeutic plan 4. Patient medication education during hospital stay and on discharge 5. Follow newly transplanted patients in the outpatient clinic every week 6. Recommend dose adjustments on medication based on changes in creatinine clearance 7. Answer questions of the clinical team	On or during patient admission, on discharge, and after discharge	Continuous	None
Bansal *et al.* (2019)	Patients	None	One-on-one	*Preadmission*: Recipient home *Postadmission*: Hospital bedside	*Preadmission*: Telephone *Postadmission*: Face to face	EMR	Always events toolkit by the NHS	Pharmacist-led ESMOS: 1. Patients are reviewed in a virtual pharmacist clinic whereby patients’ pre-existing medical comorbidities are recorded along with any high-risk medication the patient is taking 2. In the postoperative phase, close monitoring on the ward with the focus being on medicines optimization to minimize the incidence of any postoperative complications occurring 3. Work collaboratively with the multidisciplinary team	One time before admission and during the admission	Continuous	None
Bansal *et a.l* (2019)	Patients	None	One-on-one	*Preadmission*: Hospital (clinic), recipient home *On discharge*: Hospital bedside	*Preadmission*: Face to face, text messages, telephone *On discharge*: Face to face	Medical notes	Not reported	1. Provide appropriate perioperative advice and look for opportunities for medicines optimization (a reminder message or call 1 week prior to surgery was done to follow the instructions given by pharmacist) 2. Discuss with the patient’s GP about any clinical issue, with referrals sent to specialist consultants as required 3. Writing the drug chart prior to admission by a pharmacist prescriber to reduce errors that may occur 4. Medication reconciliation was undertaken to ensure that all changes were clearly documented for the patient and their GP upon discharge	Twice before admission and once on discharge	Three times	Written drug chart
Butt *et al*. (2019)	Physicians and nurses	Antibiotics	Contact with group	Hospital	Face to face	EMR	Clinical practice guidelines for antimicrobial prophylaxis in surgery (2013) by the American Society of Health-System Pharmacist	Delivered two educational and training sessions for doctors and nurses to brief and discuss the standard treatment guidelines regarding the use of antibiotics for surgical prophylaxis (duration 10–15 days)	N/A	Once	None
Elnour *et al*. (2022)	Surgeon	Antibiotics	Contact with group	Hospital	Face to face	EMR	Not reported	1. Development of SAP protocol 2. Accompany surgeons while prescribing 3. Provide structural educational activities to medical staff 4. Ensure strict adherence to the protocol	On or during patient admission	Continuous	None
Falconer *et al.* (2021)	Patients	None	One-on-one	Hospital bedside	Face to face	EMR	Primary literature review, institutional expert opinion, drug information from databases	1. Patient identification by surgery team 2. Consultation to pharmacy (medication history documentation, documentation of patient preferred pharmacy, reconciliation of home and inpatient medications for hospital admission, recommendation changes for medications as indicated, and provide patient education regarding proposed changes) 3. Assigned clinical pharmacist to perform of face-to-face inpatient consultation 4. Use standardized documentation and communication of recommendations with surgery team	On or during patient admission, on discharge	Continuous	Standardized templated note within the patient’s EMR
Fitzpatrick *et al.* (2023)	Patients	None	One-on-one	Recipient home	Face to face, telephone, written	NHS VPN, ARISE dataset	Evidence-based guideline produced in collaboration with surgical MDT, health board guidance for VTE risk assessment and procedures	1. Review electronic notes by a prescribing clinical pharmacist 2. Phone call with patients to confirm history, demographics, answer patient’s questions, and involve patient in shared decision making 3. Discuss with the surgical team to highlight or resolve perioperative medical issues 4. Individualized discharge prescription written, emailed, dispensed, and supplied to wards before patient admission	1–2 weeks before admission, 7–10 days post discharge	Twice	None
Han *et al.* (2022)	Patients	None	One-on-one	Hospital (clinic)	Face to face	Not reported	Not reported	1. A clinical pharmacist integration into the bariatric surgery clinic (as part of every patient’s preoperative clinic evaluation) 2. Each patient was scheduled for a one time 30–60 min meeting with the pharmacist prior to meeting with the surgeon 3. Obtain medication histories and provided recommendations to the patient and the team regarding perioperative medication management 4. Resolve any potential medication-related problems (e.g. medication absorption after bariatric surgery) 5. Provide medication education to the patient	Before admission	Once	None
Hyland *et al.* (2020)	Patients	None	One-on-one (patients), contact with group (rounds)	Hospital bedside	Face to face	Not reported	Primary literature	1. Comprehensive preadmission medication reconciliation 2. Preadmission patient review, VTE risk stratification, and documentation of recommendation for postoperative VTE prophylaxis 3. Verify preoperative and intraoperative medication protocol compliance or optimization for special populations 4. Review and optimization of postoperative medication orders 5. Optimize postoperative opioid-sparing multimodal analgesia strategies 6. Participate in inter-disciplinary rounds with surgical and medical teams 7. Optimize of discharge medication reconciliation and prescriptions 8. Assess and optimize the accessibility and affordability of discharge prescriptions to patients 9. Patient counseling on new medications and any medication changes at point of hospital discharge 10. Clinical support to all providers across all phases of care	Before admission, on or during patient admission, on discharge and follow-up	Continuous	None
Kwiatkowski *et al.* (2021(	Patients, medical staff, stakeholders	Antibiotics	One-on-one (patients), contact with groups (surgeons, infectious disease team, anesthesiologist, allergy department)	Recipient home (patient), hospital (medical staff and stakeholders)	*Medical staff*: face to face *Patients*: telephone	EMR	Institutional guidelines	1. Meet with stakeholders and surgery team for input and approval of intervention material 2. Delivering thorough education to the medical team 3. Telephone interviews with patients before admission 4. Update allergy status on the system 5. Notify surgeons of any necessary considerations before procedures	One week before clinic appointment	Once	EMR
Nguyen *et al.* (2020)	Patients	None	One-on-one	*First contact*: recipient home *Second and third contact*: hospital bedside	*First contact*: telephone *Second and third contact*: face to face	Not reported	Not reported	PREP pharmacist services: 1. PREP pharmacist contacted patients *via* telephone 1 week prior to surgery to obtain BPMH and medication reconciliation following a form 2. After surgery, a surgical pharmacist was provided with a handover from the PREP pharmacist for continuation of care 3. The surgical pharmacist would verify the BPMH 4. A confirmed MRF was generated by the surgical pharmacist 5. At discharge, the pharmacist prepared discharge prescriptions for the patients. The prescription is then checked and signed by the doctor	One time before admission by the PREP pharmacist, second time on admission and third time before discharge by the surgical pharmacist	Three times	MRF, EMR, handover form
Patel *et al.* (2022)	Medical staff (resident physicians and nurses)	Opioids	Contact with group	Hospital	Face to face	EMR	Best available evidence for multimodal postoperative analgesia and safe opioid use in hospitalized patients	1. Led a consensus gathering process to improve multimodal analgesia, reduce high-risk drug combinations, and lower opioid exposure 2. Provide educations to nursing staff, nursing supervisors, and orthopedics residents (responsible for postoperative orders)	N/A	N/A	None
Shang *et al.* (2021)	Patients, medical staff	Anticoagulants	One-one-one (patients), contact with group (medical staff)	Hospital bedside	Face to face	EMR	Guidelines of the ACCP and the Orthopedic Branch of Chinese Medical Association, the drug instructions	1. Conduct thrombosis and bleeding risk assessment for patients 2. Consult with physicians to formulate antithrombotic treatment protocols 3. Optimize perioperative medication regimens for special populations 4. Evaluate and optimizing the feasibility of discharge prescriptions 5. Provide consultation for medical staff and patients throughout the hospital stay 6. Provide anticoagulant-related training for medical staff every quarter 7. Follow-up the thrombosis in the first and third month after surgery	On or during patient admission, after discharge follow-ups	Continuous	None
Smith *et al.* (2018)	Patients	Opioids	One-on-one (if needed)	Recipient home	Brochure, telephone (if needed)	EMR	Brochure: developed by research team using qualitative methods, with input from patients and orthopedic clinicians *Telephone*: Motivational enhancement principles	1. Mail brochure described what patients should expect regarding opioid use and pain control after surgery (10 days presurgery) 2. A brochure was mailed that explained opioid use topics, including rationale for opioid use following surgery, opioid tapering expectations following surgery, and potential adverse effects of opioids (15 days following surgery) 3. Follow-up telephone call from a pharmacist who used motivational enhancement principles to reinforce the information in the brochure if patients filled a prescription for an opioid in the 28–90 days following surgery	One time 10 days pre admission, one time 15 days post discharge, a follow-up phone call 28 days post discharge (if needed)	Two confirmed times and one if needed	Brochure
Smith *et al.* (2023)	Patients	Anti-diabetic medications	Not reported	Not reported	Not reported	EMR	Local glycemic management model at Mayo Clinic	A clinical pharmacist-driven glycemic management model is utilized for well-controlled subset of the population	On or during patient admission	Continuous	None
Surgery and pharmacy in Liaison (SUREPILL) Study Group, 2015	Patients	None	One-on-one	Hospital bedside	Face to face	EMR and patient history taking	Hospital protocol	1. Medication reconciliation 2. Consultation with the patient using a standard questionnaire 3. Review medication chart and optimizing medications when needed 4. Perform interventions with liaison with the physician 5. Weekly patient meetings (when possible) 6. Review discharge medications 7. Patient counseling about the medications	On or during patient admission, on discharge	Continuous	None
Tong *et al.* (2022)	Medical staff	TPN	No direct contact	N/A	N/A	EMR	ESPEN, American Society for Parenteral and Enteral Nutrition, Chinese Medical Association for Parenteral and Enteral Nutrition	Pharmacist-led standardization program for TPN administration: 1. The pharmacists established an evaluation standard for perioperative TPN 2. Subsequently, a computerized TPN management system was developed and applied, including a prescription management system and evaluation software, to identify and reduce unsuitable prescriptions 3. For continuous improvement, the inappropriate prescriptions were sorted out and feedback was provided to the doctors for confirmation	N/A	N/A	None
Van Prooyen *et al.* (2023)	Patients	None	One-on-one	Hospital bedside	Face to face	EMR	Institutional protocol created utilizing guideline recommendations, primary literature review, drug information databases, and the team’s experience and expertise	1. Complete and document medication history 2. Review home medication list for needed postsurgical medication changes 3. Create a discharge medication plan based on a defined protocol 4. Document the recommended discharge medication plan in a consult note (prior to discharge, the provider reviewed the note and performed and signed the discharge medication reconciliation) 5. Patient education outlining the discharge medication plan	Postoperative day 1	Once (patients), as needed (physician)	Consult notes on EMR
Wang *et al.* (2020)	Patients, surgeons, anesthesiologist	Antiemetics	One-on-one (patients), contact with group (surgeons, anesthesiologists)	Hospital bedside	Face to face	EMR	ASA and hospital protocol	1. Review of patients’ medical records 2. Feedback of current PONV and inappropriateness to surgical team 3. Share and discuss evidence (education outreach) 4. Maintenance of highlighting of PONV optimization, timely discussion with surgeons and anesthesiologists, and daily review of patients	On or during patient admission	Continuous	None
Wang *et al.* (2023)	Patients	Analgesia	One-on-one (patients), contact with group (rounds)	Hospital bedside	Face to face, written	EMR	EUSEM guideline, ERAS protocol, and expert consensus	1. Preadmission assessment 2. Preoperative intervention on high-risk patients (MDT) discussions for multimodal pre-emptive analgesia plan, patient education perioperatively 3. Postoperative (monitoring and analgesia adjustment, MDT rounds, case discussions, using WeChat workgroups to communicate with patients, ADRs monitoring) 4. Discharge review and management	1. Within 48 h of preadmission 2. Within 24 h after preoperative 3. 24 h postoperative 4. 24–48 h before discharge	Four times	Brochures, WeChat workgroups
Yang *et al.* (2019)	Patients	None	One-on-one (patients), contact with group (medical team)	Hospital bedside	Face to face	Not reported	Not reported	1. Direct patient care and medication management during hospitalization 2. Review medication regimens 3. Resolve medication-related problems 4. Medication reconciliation 5. Answer drug information questions 6. Therapeutic drug monitoring 7. Make therapeutic recommendations 8. Patient education	On or during patient admission	Continuous	None
Zhang *et al.* (2021)	Patients, medical staff	PPI	Contact with group (rounds and educational sessions)	Hospital bedside (rounds), hospital (educational sessions)	Face to face	EMR	*Martindale*: The Complete Drug Reference (39th), New Materia Medica, drug instructions, American Society of Health-System Pharmacists criteria and expert consensus	1. Participate in daily medical rounds and clinical duties 2. Targeted educational interventions for medical staff	On or during patient admission	Continuous	None
Zheng *et al.* (2022)	Patients	None	One-on-one	Hospital bedside	Face to face	Not reported	Primary literature on ERAS protocols	1. Review medications orders and history of patients within 24 h of admission 2. Interview patients to obtain medications history 3. Check the pre- and postoperative medication orders for discrepancies from the ERAS options 3. Within 24 h before discharge, discharge prescriptions were checked 4. Monitor and consider discrepancies and discuss with surgeons	On or during patient admission, at discharge	Continuous	None
Zhou *et al*. (2016)	Patients, surgeons, nurses	Antibiotics	Contact with group (rounds, educational sessions)	Hospital bedside (rounds), hospital (educational sessions)	Face to face (rounds, educational sessions), written (handouts)	EMR	Clinical guidelines	1. Participate in ward rounds and making drug treatment plans 2. Communicate immediately with surgeons when irrational antibiotics were prescribed 3. Provide educational sessions and handouts about antibiotic prophylaxis for medical teams (physicians and nurses) 4. Extract the medical records and assessing the responsible use with the help of electronic auditing system 5. Report the categorized data on irrational use of prophylactic antibiotics to leadership of cardiothoracic surgery department every week	On or during patient admission	Continuous	Handouts, electronic auditing system
Zhou *et al.* (2023)	Patients, surgeons, nurses	Antibiotics	Contact with group (rounds, lectures), one-on-one (clinic)	Hospital bedside (rounds), hospital (clinic), hospital (educational sessions)	Face to face (rounds, lectures), written (brochures)	EMR, outpatient clinic revisit records, telephone follow-up data	NHFPC guidelines, published official documents	1. Participate in daily rounds in orthopedic ward and developing plans for antibiotic therapy with physicians 2. Discuss with orthopedic physicians to formulate perioperative antibiotic prophylaxis norms for internal fixation 3. Instructing the implementation of anti-infection plan by nurses 4. Distribute brochures on antibacterial drugs for healthcare providers 5. Provide special lectures on rational use of antibiotics every quarter and discussing and communicating the problems arising from application of antibiotics 6. Follow-up the infection complications and readmission of patients at 6 months postoperatively	During admission and on clinic follow-up (unclear when appointment was)	Continuous	Brochures

ACCP, American Association of Chest Physicians; ADR, adverse drug reaction; ARISE, Arthroplasty Rehabilitation in Scotland Endeavor; ASA, American Society of Anesthesiologist; BPMH, best possible medication history; CMS, Centers for Medicare & Medicaid Services; DEPICT, Descriptive Elements of Pharmacist Intervention Characterization Tool; GP, general practitioner; EMR, electronic medical record; ERAS, enhanced recovery after surgery; ESMOS, Enhanced Surgical Medicines Optimization Service; ESPEN, European Society for Clinical Nutrition and Metabolism; EUSEM, European Society for Emergency Medicine; MDT, multidisciplinary team; MRF, medication reconciliation form; N/A, not applicable; NHFPC, National Health and Family Planning Commission; NHS, National Health Service; PONV, postoperative nausea and vomiting; PPI, proton pomp inhibitor; PREP, PeRiopErative and Prescribing; SAP, surgical antimicrobial prophylaxis; TPN, total parenteral nutrition; VPN, Virtual Private Network; VTE, venous thromboembolism.

### Description of pharmacist-led interventions

**Figure 2.**
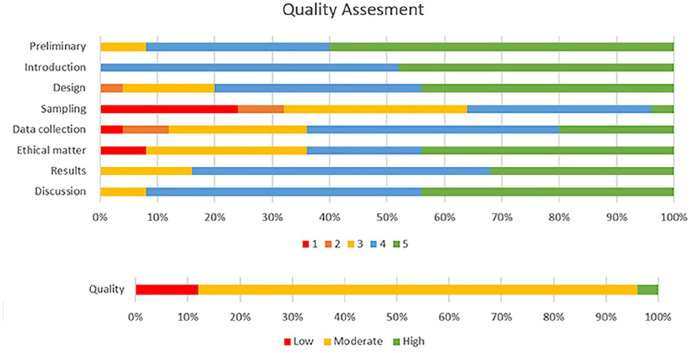
Stacked bar chart representing quality of included studies.

**Figure 3.**
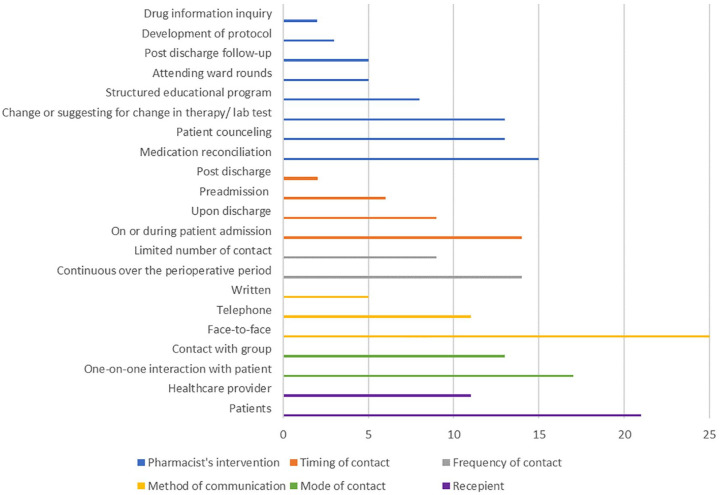
General characteristics of the interventions performed by the pharmacists.

Table 2 and Figures 2 and 3 describe the pharmacist-led interventions in each study as described by DEPICT-2.^28^

#### Recipients of the intervention

Fourteen studies (56%) reported that patients were the target of the implemented pharmacist-delivered services,^33–35,38–40,44,45,47,50–54^ while seven studies (28%) reported that patients alongside medical staff were the recipient of the intervention.^32,36,41,43,46,48,49^ Four studies (16%) reported that the pharmacist intervention was intended for medical staff, mainly surgeons with or without other healthcare providers such as nurses or anesthesiologists.^37,42,55,56^

#### Setting where recipient received intervention

All studies, except for one,^39^ reported the settings where the intervention took place. The intervention was delivered at hospital bedside in nine studies (36%),^33,35,40,41,43–45,47,54^ while another nine (36%) reported other settings alongside the hospital bedside such as hospital clinic,^32^ recipient’s home,^51,53^ hospital in general (refers to interventions that included educational session),^37,46,48^ or a combination of them.^49,50^ Two studies (8%) only included educational initiatives and were conducted in the hospital,^55,56^ while one study included both delivering education in the hospital and interviewing patients in their homes.^36^ Two interventions took place only at the recipient’s home^38,52^ and one in a hospital-based clinic.^34^

#### Communication with recipients

Most interventions (*n* = 9; 36%) were conducted through one-on-one contacts with the patients either in the clinic or during the medication reconciliation process,^33,34,40,47,50–54^ while five interventions (20%) were contact with group which was mostly either clinical rounds or educational sessions.^37,46,48,55,56^ Eight interventions (32%) included a combination between one-on-one and contact with group.^32,35,36,41,43–45,49^ Smith *et al.*^38^ reported that they provided one-on-one contact but only if needed, while Tong *et al.*^42^ reported that there was no direct contact as the intervention was to develop TPN software. Only one study did not report the mode of contact with the recipients.^39^

The method of communication was largely (*n* = 13; 52%) through face-to-face contacts with patients or other healthcare providers,^33–35,37,40,41,43,45–47,54–56^ while nine studies (36%) encompassed telephone,^32,36,51,53^ written contacts,^44,48,49^ or both^50,52^ beside the face-to-face communication. It is worth pointing out that the studies that used a combination method of contact was mainly through virtual contact with the patient before admission to reconcile the medications (as it might be too late to make necessary changes upon admission), followed by in-person contact after admission. Smith *et al.*^38^ included written communication *via* mailed brochure and telephone if needed. Four studies did not involve direct contact with the patient as the intervention targeted healthcare providers^37,42,55,56^ and one study did not report whether there was a direct patient contact or not.^39^

Of the 25 included studies, 14 (56%) demonstrated a continuous provision of the pharmacist services over the perioperative period,^32,33,35,39,41,43,45–49,51,54,56^ while nine (36%) studies reported that the frequency of contact was limited to a specific number of times (ranging from one to four times).^34,36,38,40,44,50,52,53,55^ Among the former, some studies (*n* = 6; 24%) reported that the services were only delivered on or during patient admission,^38,43,45,46,48,56^ while others reported that the intervention was initiated before admission (*n* = 1; 4%),^51^ extended after discharge (*n* = 6; 24%),^33,41,47,49,54^ or both (*n* = 1; 4%).^32,35^ Among the latter, the timing of the pharmacist intervention was variable including delivery of services only preadmission,^34,36^ preadmission and on discharge,^50,53^ preadmission and postdischarge,^38,52^ preadmission, during admission (once each preoperatively and postoperatively) and on discharge,^44^ and postoperative.^40^ For studies that involved contacting the patient before admission, it was done 48 h to 2 weeks beforehand, whereas for studies that involved contacting the patient after discharge, it took place 7–15 days of discharge.

#### Pharmacist action(s)

Most studies conducted a multifaceted pharmacist-led intervention (Figure 3 and Table 2). This involved clinical pharmacy services during admission such as attending ward rounds, reviewing patient records, medication reconciliation, answering drug inquiries, and patient counseling. Additionally, some interventions incorporated medication reconciliation before admission by contacting the patient (in a physical or virtual clinic) or the patient’s general practitioner or community pharmacist as part of the patient preparation for the surgery. This could reduce discrepancies upon admission and allow the pharmacist to advise on holding/starting/adjusting medications in the appropriate timeframe as it could be too late if it was stopped on admission (e.g. anticoagulation medications should be stopped 3–7 days before surgery). Some interventions also incorporated follow-up appointment with the patient postdischarge in a virtual of physical clinic, this was particularly important in patients who underwent surgeries that might require long-term use of medications (e.g. transplant or cardiothoracic).

Other interventions comprised pharmacist-led structured educational programs (either alone or in addition to the clinical pharmacy services) delivered to various healthcare providers (mainly surgeon and nurses).^36,37,41,46,48,49,55,56^ Pharmacists also developed protocols (e.g. surgical antimicrobial prophylaxis) or TPN software to assist surgeons during their practice, in some of the studies the pharmacist was also responsible for implementing and ensuring that the protocols were accurately followed.^36,37,42,56^ Conducting internal audits and report findings was another intervention that emerged among the included studies, in which findings from the audit were utilized to provide individualized or group feedback.^48^ Referral to other healthcare providers has also been identified among the included studies.^50^ The level of detail regarding the development, structure, and processes of these interventions was limited.

#### Source of guide for intervention

Twenty out of the 25 included studies (80%) furnished some information on the basis for developing the implemented intervention (Table 2). The primary source of guide for the pharmacist-led interventions was clinical guidelines^32,42,43,48,55^ and institutional protocols.^36,39,40,52,54^ Along with the guidelines, some studies employed other sources such as drug instructions,^41,46^ expert consensus,^33,44^ or published official documents.^48^ Three studies based the intervention on primary literature search.^35,37,47^ Bansal *et al*.^51^ reported the use of the previously developed ‘Always Events’ toolkit. Smith *et al*.^38^ reported that the brochures were developed by the research team using qualitative methods with input from patients and orthopedic surgeons, while the telephone calls were based on the motivational enhancement principles.

#### Materials that supported the intervention

Only nine studies (36%) reported that they utilized materials to support the intervention (Table 2). This mainly included forms/notes (whether impeded in the electronic medical record or not) to facilitate communication between healthcare providers,^33,36,40,50,53^ and brochures/handouts to patients^38,44^ or surgeons and nurses.^48,49^ One study used WeChat work groups to improve communication with patients.^44^

### Outcomes of the pharmacist-led interventions

#### Length of stay

**Figure 4.**
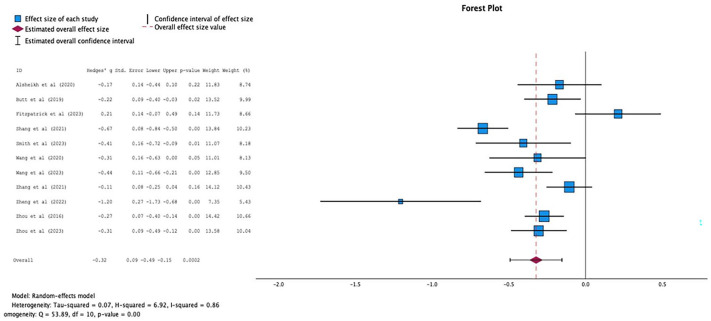
Forest plots of intervention effects on length of stay (LOS).

**Figure 5.**
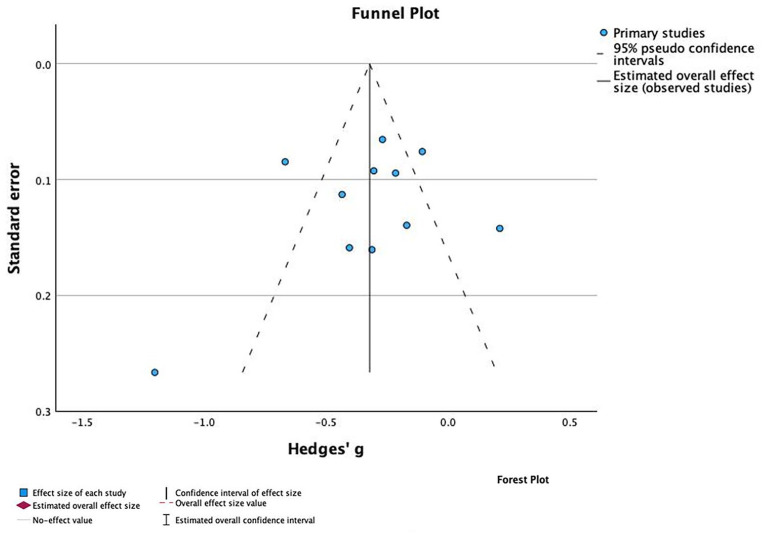
Funnel plot to assess the publication bias for studies assising phamacist impact on length of stay (LOS).

Of the 21 studies that reported data on the inpatient LOS, only 11 reported adequate data for meta-analysis.^32,39,41,43,44,46–49,52,55^ These studies demonstrated a SMD (*n* = 4905 patients) favoring the pharmacist intervention group of −0.09 days (95% CI −0.49 to −0.15) with considerable heterogeneity (*I*^2^ = 86%, overall effect *p* = 0.0002) (Figure 4). There was no evidence of publication bias based on the symmetrical distribution of studies along the funnel plot’s null line and Egger’s regression test (*p* = 0.995) (Figure 5).

Among studies that were not included in the meta-analysis, only half of the studies showed significant decrease in the LOS.^36,37,50,51,56^ Studies that did not show statistical significance mainly shared one or more of the following factors: (1) the intervention comprised only one pharmacist service (mainly medication reconciliation); (2) the intervention was not continuous throughout the perioperative period; (3) there was a lack of preadmission contact with the patient to optimize drug therapy prior to admission.

#### All-cause readmission

**Figure 6.**
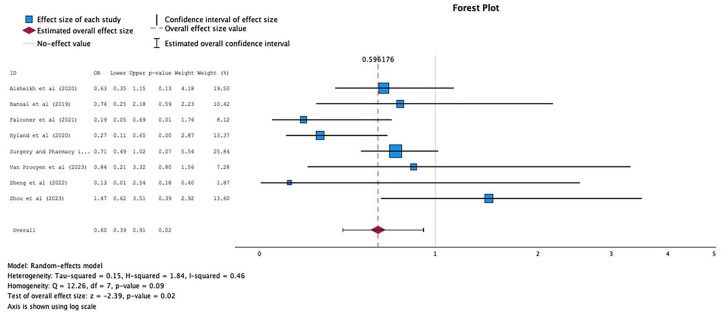
Forest plots of intervention effects on all-cause readmission.

**Figure 7.**
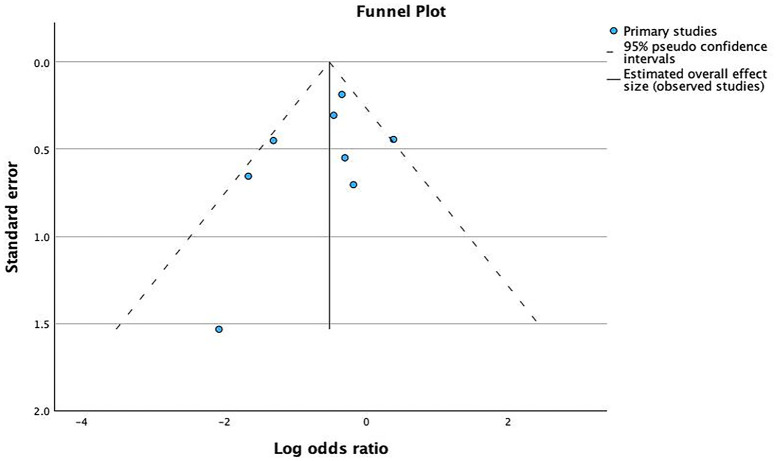
Funnel plot to assess the publication bias for studies assising phamacist impact on all-cause readmission.

Eight of nine studies that measured all-cause readmissions were pooled. Seven studies reported this outcome at 30 days,^32,33,35,40,45,47,50^ while one each reported at 3 and 6 months.^49,54^ Although only two studies showed statistically significant reductions in readmissions,^33,35^ the ORs and CIs for studies with higher weight were only marginally nonsignificant^32,54^; therefore, the overall effect was significant. The pooled analysis across all interventions (*n* = 3271 patients) showed some significant difference between the intervention and usual care (OR 0.60; 95% CI 0.39–0.91; Figure 6). Evidence showed moderate heterogeneity (*I*^2^ = 46%) in this outcome. The funnel plot revealed no marked asymmetry supported by Egger’s test value of 0.905; hence, no evidence of publication bias (Figure 7).

Only one study was not included in the meta-analysis. The readmissions in Yang *et al*.^45^ were 0% in both groups at 7 and 30 days. Overall, studies that showed significance implemented a multicomponent continuous intervention that involved structured discharge counseling or postdischarge follow-ups.

#### Other clinically important outcomes

Only five studies investigated other clinically important outcomes, with one study exploring two endpoints of interests (Table 1).^33,38,40,47,52^ Three outcomes emerged from included studies which are physical or virtual (*via* phone calls or email) unplanned outpatient visits,^33,38,47^ ED visits,^38,40^ and any healthcare encounter.^52^ None of the included studies examined the impact of pharmacist interventions on mortality.

Smith *et al*.^38^ distributed educational brochures to patients without direct contact with the pharmacist, findings showed a slight increase in unplanned outpatient visits (face to face, telephone, or e-mail). Similarly, Falconer *et al.*^33^ focused on patients undergoing bariatric surgery and showed a significant increase in outpatient medication-related phone calls from 31% to 39% (*p* = 0.04).^33^ In contrast, Zheng *et al*.^47^ included patients undergoing orthopedic surgeries and showed an insignificant decrease in unplanned outpatient visits within 30 days of discharge (*p* > 0.05).^47^

While Van Prooyen *et al*.^40^ showed a slight increase in ED visits within 30 days from 2.4% to 5.9% (*p* = 0.28),^40^ Smith *et al.*^38^ revealed an insignificant decline in the number of ED visits. A UK-based retrospective study that focused on orthopedic patients showed a significant decrease in 9-day any healthcare encounter (*p* = 0.03).^52^

### Quality of evidence

The scores ranged between 25 and 36, with a median score of 32.16. The review indicated that the majority of studies (*n* = 21; 84%) were of moderate quality, while three (12%) showed low quality, and one (4%) was identified as a high-quality article. The key limitations centered on sampling, data collection, and ethical matters (Figure 2).

## Discussion

This systematic review and meta-analysis is the first to examine the effectiveness of pharmacist-led interventions in the perioperative settings on clinically important outcomes. It also provides a comprehensive summary of the characteristics of the pharmacist-provided interventions. It is evident from our systematic review that research focusing on the impact of pharmacist interventions during the perioperative period has been rapidly accelerating since the year 2019.

### Healthcare resource utilization

This review has shown better outcomes in favor of pharmacist-provided interventions. We found a substantial reduction in the LOS (SMD −0.09 days; 95% CI −0.49 to −0.15) and all-cause readmissions (OR 0.60; 95% CI 0.39–0.91). Previous research showed mixed findings in relation to the impact of pharmacist-delivered services on the LOS. For example, two meta-analyses that implemented pharmacist-led medication reconciliation and medication-related interventions demonstrated no effect on the hospital LOS.^57,58^ Conversely, implementation of a ward-based pharmacist who performed multiple services shortened the LOS by (SMD −1.74 days; 95% CI −2.76 to −0.72).^59^

Less variation has been noted among studies that explored the influence of pharmacist interventions on the readmission rate. In line with our findings, different pharmacist-led interventions have shown significant reduction in readmission rate in various settings and patient populations.^57,59−62^ However, most studies concluded that the quality of evidence is low and that there is lack of high-quality RCTs.

It also is important to note that some of the previously discussed studies showed improvement in one endpoint but not the other. Interventions have the potential to create trade-offs between outcomes. Reducing LOS might increase concerns for readmission risk or shifting costs of care to the outpatient setting.^21,63^ Nevertheless, new evidence suggests that there is no correlation between readmission rates and LOS rates.^64,17^ In fact, it was proposed that shorter LOS might be associated with lower readmission rate if the patients was not discharged prematurely, and if there was emphasis on ambulatory operations throughout this time and on easier access to outpatient appointments, especially within 7 days of discharge.^65^ Therefore, evidence emerging from our review is pivotal as it confirms that pharmacist interventions in perioperative settings have positive impact on both LOS and readmission which highlights the importance for comprehensive multidimensional interventions that are continuous throughout the perioperative period.

Pooling data was infeasible for other relevant outcomes (unplanned outpatient visits, ED visits, and any healthcare encounter); however, findings from these studies were conflicting. Thus, at present, the strength of evidence is insufficient to reach firm conclusions on the role of pharmacist on these outcomes. It is worth noting that this kind of evaluations often requires larger sample sizes due to high variability.^66^

### Components of the pharmacist interventions

Most studies implemented multicomponent interventions including an array of clinical services (e.g. medication reconciliation, attending rounds, patient counseling) alongside other interventions such as education of health professionals, developing clinical pathways, and referral to other healthcare providers. Pharmaceutical care combined with education was the most common intervention strategy amongst included studies. This is consistent with a previous review focused on people living with HIV/AIDS. The study concluded that pharmacist care improves a wide range of clinical outcomes and that the most common intervention type in this setting is education combined with pharmaceutical care.^67^ It hence is imperative to reinforce the importance of educational interventions as previous research highlighted that education has been integrated into each intervention strategy and is seen as a key to improving multiple clinical outcomes.^68,69^

Nevertheless, single component interventions (most commonly medicines reconciliation) were also relatively common in our review. Although we did not conduct a subgroup analysis, individual studies with multifactorial interventions showed a trend toward significance as compared to single component interventions. This finding is consistent with previous research suggesting that interventions consisting of intermingled components are associated with improvement in various medication safety and patient outcomes as compared to a single isolated intervention. For instance, a meta-analysis focusing on patients transitioning out of the intensive care unit showed that multicomponent interventions led to a fourfold reduction in deprescribing on hospital discharge as compared to twofold in the single component arm.^58^ Similarly, another meta-analysis illustrated that various combinations of interventions were associated with lower risk of injurious falls compared with usual care in older adults.^70^

While studies that focused on pharmaceutical care provided relatively sufficient details on the characteristics of the intervention, studies presenting other types of interventions provided a considerably varied level of details and was generally lacking which could hinder the reproducibility of these interventions. This long-standing issue has been extensively discussed in the literature.^69,71–75^ Therefore, our findings suggest that, to some extent, there is progress in characterizing pharmaceutical care interventions in recent years especially that most of the included studies are recent. Yet, describing other kinds of interventions is still lacking.

### Timing and frequency of the pharmacist interventions

A small majority of the included studies employed a continuous intervention throughout the perioperative period. By looking at each individual study, it was evident that those studies mostly resulted in significant findings as opposed to studies that were limited in terms of timing or frequency.

Additionally, studies that showed a significant reduction in the LOS shared the fact that most of them incorporated a preadmission contact with the patient (mostly 1–2 weeks preadmission) to reconcile medications and optimize drug therapy prior to admission. Nonetheless, studies that only implemented a medication reconciliation program upon admission mostly did not yield any meaningful improvements. This contrasts with a previous meta-analysis that proved pharmacist-led medication reconciliation services as an effective standalone intervention.^57^ This suggests a plausible temporal relation that is specific to the perioperative settings, between the reconciliation and its effectiveness. Patients who are scheduled for surgery are usually admitted 1 day before the surgery.^76^ This means that the patients are not reviewed by a pharmacist until the day of admission for surgery. By this time, it is often too late to make any significant changes to perioperative drug management and on occasions patients may not be seen on the morning of scheduled surgery.

Similarly, studies that showed significance regarding the readmission rate generally implemented a continuous intervention that involved structured discharge counseling or postdischarge follow-ups. This substantiates previously published evidence that multicomponent interventions that involve high-quality discharge planning and structured planned postdischarge support decreases readmission rate.^23,65,77,78^ Notably, current literature also suggests that obtaining an accurate preadmission medication history have great potential to reduce harm, as they can propagate throughout a patient’s hospitalization and after discharge.^23,79^

### Development of the pharmacist interventions

Our findings suggest a paucity of data from all included studies on the basis of the intervention development which could hamper the application of these interventions by other researchers and policymakers. Whilst it was noted that most reviews relied on local or international guidelines as the scientific basis for the development, no further details were provided. Furthermore, only one study reported a theory underpinning intervention development which was the motivational enhancement theory without any further detail on how it was applied.^38^ There is an accumulation of evidence that theory-informed interventions are more likely to yield positive and sustainable outcomes compared to pragmatic approaches.^72,80–82^ Similarly, Glasgow and Linnan^83^ suggest that the use of theory ‘results in more powerful interventions’. The absence of reporting on the theoretical underpinnings of the included interventions limited our commentary on its effectiveness. Moreover, reporting the impact of theory-driven interventions in this area remains unknown. It has also been explicated by Michie *et al*.^84^ that even if the theory does not confer a positive effect on outcomes in favor of the intervention, it helps to clarify what does and does not work in an intervention.

### Strengths and limitations

To the best of our knowledge, this is the first systematic review and meta-analysis to evaluate the available literature on the characteristics and impact of pharmacist interventions on clinically important outcomes in perioperative settings. The DEPICT-2 tool was used for systematic extraction and analysis of the intervention’s core components among included studies, which eliminated the rater effect and, hence, provided more consistency to our results.^28^ The protocol was prospectively registered on PROSPERO.

There are some limitations. First, the study was limited to English and Arabic language publications; this may have excluded relevant references published in other languages. Second, included studies were restricted in terms of the sample size and follow-up duration which is required to detect the investigated outcomes comprehensively. Third, there was considerable heterogeneity in the data from the meta-analysis; however, this limitation is expected in multifactorial complex interventions. Fourth, although we searched Google Scholar to assist in locating nonindexed articles, using Google Scholar alone may not be sufficient.^85^ Hence, there may be gray literature that was not identified in our search.

### Future directions

Findings from our study suggest that multifaceted pharmacist-delivered interventions are promising in improving clinically important outcomes, primarily LOS and readmission, in perioperative settings. Therefore, developing and implementing interventions tailored specifically to these settings should be a policy and practice priority, being particularly pertinent to the complexity of surgical patients and the significant differences in the perioperative environment and infrastructure. Future research should therefore address the contextual factors (e.g. medication discrepancies, medication management perioperatively, unsafe discharge) contributing to negative outcomes across the whole surgical care pathway.^14^

It is noteworthy that none of the included studies investigated the impact of pharmacist interventions on mortality even though mortality is considered an integral outcome measure to assess the quality and efficacy of hospitals and healthcare systems.^16–18^ We therefore encourage future researchers to consider investigating the effect of pharmacist-led interventions on mortality to have a holistic view of the pharmacist impact on clinical outcomes. We also encourage researchers to conduct a comprehensive cost assessment (e.g. cost-effectiveness analysis) of pharmacist-led interventions in the perioperative setting to inform evidence-based policy decisions. This will subsequently assist policymakers in developing targeted strategies and allocating resources.

The current study also highlighted the lack of theory-driven interventions. Thus, there is a need for several sufficiently powered randomized studies of a theoretically derived intervention aiming to improve clinical outcomes in the perioperative settings. The Medical Research Council Framework of Complex Interventions in the United Kingdom advises the use of theory and exploratory studies to identify barriers to change while developing complex interventions.^86^ It is imperative that future interventions utilize behavioral theories to strengthen the impact and ensure the sustainability of interventions.^87^ This is of extreme importance as the follow-up duration for studies included in our review was short, which could raise concerns regarding the long-term effect of these interventions. Additionally, the various interacting components in behavior change research makes it challenging to identify the active, effective components within interventions and for others to replicate them.

On that account, it is of paramount importance that detailed descriptions of the interventions, in terms of structures, processes, and outcomes, are included in publications to allow them to be reproduced and for readers to consider the studies within the context of their own practice. We therefore endorse the use of the DEPICT-2 tool to structurally describe the intervention of interest in pharmacy practice research.^28^

Furthermore, it should be noted that none of the studies investigated prescribing (either independently or collaboratively) as part of the clinical pharmacy practice. Pharmacist prescribing showed promising results as it offers a wide range of benefits such as quicker and more efficient access to medicines for patients, a reduction in physician workload, and enhanced professional satisfaction.^88,89^ These favorable outcomes might be amplified in perioperative settings due to additional barriers that exist in surgeons’ practice such as logistics, time, and knowledge about medications, which might affect their ability to prescribe safely.^90,91^ Therefore, pharmacist prescribing in perioperative settings could be a potential area of work in the future.

## Conclusion

The results of this meta-analysis indicate that pharmacist-led interventions in perioperative settings decreased the LOS and readmissions. However, the effect on other healthcare utilization endpoints is inconclusive based on the current body of evidence. There is some evidence that multifaceted complex interventions that occurred throughout the perioperative period, starting from preadmission medication reconciliation to comprehensive discharge plan, are more likely to yield positive impact but this is generally of low quality and insufficient volume. Findings from this review help policymakers design appropriate theory-informed pharmacist interventions in perioperative setting by keeping in view their available resources.

## Supplemental Material

sj-docx-1-taw-10.1177_20420986241260169 – Supplemental material for Interventions and impact of pharmacist-delivered services in perioperative setting on clinically important outcomes: a systematic review and meta-analysis Supplemental material, sj-docx-1-taw-10.1177_20420986241260169 for Interventions and impact of pharmacist-delivered services in perioperative setting on clinically important outcomes: a systematic review and meta-analysis by Lina Naseralallah, Somaya Koraysh, Bodoor Aboujabal and May Alasmar in Therapeutic Advances in Drug Safety

## Declarations

**Ethics approval and consent to participate:** Not applicable as we conducted a systematic review that did not involve participants or patients.

**Consent for publication:** Not applicable as we conducted a systematic review that did not involve participants or patients.

**Availability of data and material:** Collated data from the study is available from the authors on request.
